# Flexural capacity of eco-friendly reinforced concrete beams

**DOI:** 10.1038/s41598-023-47283-6

**Published:** 2023-11-17

**Authors:** Nagib N. Gerges, Camille A. Issa, Nariman J. Khalil, Lara Abdul Khalek, Serge Abdo, Yehia Abdulwahab

**Affiliations:** 1 University of Balamand, Balamand, Lebanon; 2 Lebanese American University, Byblos, Lebanon

**Keywords:** Engineering, Environmental impact

## Abstract

In the construction industry, concrete is the most utilized building material. It is produced from different natural resources such as sand and gravel, as well as cement. The production of concrete is causing harm to the environment, yet its use became a necessity. To solve this humongous environmental challenge, many researchers devoted a considerable effort to partially replacing concrete mix components with waste material derived from glass, plastics, aluminum, wood ash, construction and demolition wastes, or tires. In the current study, a novel effort was conducted to incorporate all the above-mentioned wastes in a concrete mix design and to assess its performance. Five recycled mix designs were explored and based on the concrete mechanical properties derived, an optimum mix was realized. The optimum mix incorporated the following waste percentages: 2% crumb rubber (CR) partially replacing sand, 20% powdered glass (PG) partially replacing sand, 50% recycled concrete aggregates (RCA) partially replacing coarse aggregates, and the addition of 0.5% plastic. The optimum recycled mix was utilized to cast a real-life-size reinforced concrete beam which was compared to a normal mix beam.

## Introduction

One of the most critical global problems to be solved is pollution with all its forms, which is drastically affecting the environment. Land pollution is one type of pollution which includes the desecration of land and contamination of earth due to many pollutants such as wastes, chemicals, sewage, and others. Landfills, by definition, are the dumping of wastes in soil. They are created to be a good solution of discard waste. The wastes dumped include decomposable solid and organic wastes, as well as wastes that are hard to be decomposed like plastics, glass, construction and demolition wastes, industrial wastes, and others^1^.

With the dawn of the twentieth century, plastic evolved into being an essential material being utilized into various products such as bottles, gallons, or bags, etc. This caused the increased in the production of plastic materials due to the increase in demand. According to EPA (Environmental Protection Agency), municipal solid wastes (MSW) contain a major category for plastic about 12.2%^2^. Disposing of plastic has been an important dilemma for years. Dumping plastic into the environment recklessly lead to the pollution of the land. Plastics in general are not biodegradable, with the presence of sun and environmental factors, plastics degrade into smaller particles that eventually remain in environment without further degradation. According to Jiang, one plastic bottle takes about 1000 years to be degraded^3^. The solution of Reduce Reuse Recycle (3Rs) was a great idea whose implemented concept was to decrease the impact of disposal of plastic. But although recycling of plastic helped in reducing plastics disposal to the environment by about 29%, yet plastics are still being delivered to landfills in about 18.5% of the whole MSW^2^. This means that, land pollution problem due to plastic is still unsettled.

Similarly, glass is a material used on daily basis in many shapes and forms. Its demand is increasing constantly, especially in construction, medical field, and others. Glass is considered being a sustainable material since it could be used repeatedly, which means that its lifespan is greater than that of plastic. Yet, after excessive use, it is eventually disposed of in landfills. Same as plastic, glass constitutes about 5% of the MSW. Although it is 100% recyclable, only 31.3% of the total amount of glass found is being recycled^4^.

Aluminum is another industrial product that ends up in landfills. It is used in cans, food packaging, construction, industries, and others. Aluminum has the capacity to react with various materials in landfills such as alkaline water releasing large amounts of energy^5^. It can be used as a basic component of fireworks and explosives as it is a good conductor of heat and electricity^6^. Another alternative is to utilize the aluminum waste in construction.

According to Azevedo et al.^7^, motor vehicles are going to reach 1.2 billion representing almost 5 billion tires that are disposed by end of year 2030. Every malfunctioning rubber tire is discarded—not recycled or treated—into landfills. Other than their complex degradation, tires have the potential to burn easily in landfills causing the discharge of numerous toxic chemicals to the soil and air. An alternative of discarding tires in landfills is to utilize crumb rubber (CR) in concrete mix design. CR is produced by shredding and grinding disregarded tires into very small particles.

Construction and demolition wastes (CDW) are materials formed from residues and debris during the concrete construction or demolition process. EPA promotes and identifies that CDW are resources that might be utilized in new construction developments, thus abolishing the necessity for virgin materials to be extracted and processed^8^. These wastes are dumped in landfills as part of the MSW. The availability of these wastes in landfills has a tremendous negative ecological impact. According to Chemical Engineering Transaction (CET)^9^, the construction and demolition wastes disposed in landfills cause increased emissions of greenhouse gases (GHG) with a yearly emission level of up to 39% of CO_2_ per year. Add to that locally, the Beirut Blast of August 4, 2020, has left Lebanon with 800,000 tons of CDW^10^. Recycled concrete aggregates (RCA) generated from CDW is being used to replace natural aggregates in concrete.

Wood ash (WA) is the deposit that originates from wood combustion in chimneys, oven bakeries, industries, and others. WA has been dumped recklessly or at best been sent to landfills as waste. WA could be recycled and used in concrete mix as indicated by several researchers^11^.

As a result of the various similarities in chemical and physical properties with concrete components, incorporating these wastes in concrete mix design has yielded promising results. The main objective of this research is to combine wood ash, crumb rubber, shredded plastic bottles, powder glass, recycled concrete aggregates, and fine size aluminum flakes in one concrete mix realizing a concrete mix that is adequate for structural purposes. To achieve this objective, five different concrete mix designs incorporating waste materials in variable percentages replacing a certain component of the mix was explored. In this study, WA was used to partially replace cement and sand, CR and PG were used to partially replace sand, RCA to partially replace coarse aggregates, and shredded plastic and aluminum were used as additives in concrete. The mechanical properties of these mixes were compared to a control mix and finally the optimum recycled mix was utilized to construct a real-life size reinforced concrete which was tested and compared to a normal mix beam.

## Background

Pollution has been devastating nature all around the world. It has expanded its roots in every natural resource available in the environment to form land pollution, air pollution, and water pollution. According to Traceability and Verification System for Health Products (TRVST)^12^, 91% of all methane and carbon dioxide emissions to the atmosphere are results of open landfills. Air pollution has reached high levels exceeding World Health Organization (WHO) limits, where 91% of population live in areas of high contamination of air^13^. All these issues are men made. Since none of these issues are being resolved, many serious health problems and diseases arose affecting human and environment where levels of carcinogenic materials are increasing drastically each year. The introduction of waste materials in concrete has been considered a good alternative for dumping wastes in landfills or recycling of these wastes. Although recycling of waste reduced the presence of these wastes in landfills, it required a lot of energy to be achieved producing toxins and impurities. With the main objective of this research being a sustainable environment, the recycling was not considered as an ideal solution. The main advantage of the production of eco-friendly concrete by replacing certain percentages of natural resources, reduced the extraction of raw materials and the production of cement material, as well as the presence of wastes in landfills without releasing any toxic materials or gases to the environment.

Aluminum is a material used in several fields and specifically construction. Aluminum waste is present in domestic wastes, industrial wastes, or even in production of aluminum. In domestic wastes, it is available as 1.8% of MSW^14^. Diverse types of aluminum materials are dumped in landfills. A significant percentage of dumped aluminum material that is being recycled are reused. Yet according to EPA, 2.7 million tons of aluminum materials are present in landfills^14^. Waste produced during production of aluminum are of different forms depending on the amount of aluminum that it contains. Aluminum flakes are one example of aluminum waste product that is formed after drilling aluminum surfaces like windows and doors. This type of waste is eventually disposed of in landfills. Despite the origin of aluminum waste, when this type of waste is dumped in landfills, chemical reactions can occur specially in presence of alkaline water where toxins and noxious odors and gases are formed^15^. Some research was performed on including various aluminum materials in concrete mix design to reduce aluminum made waste products from landfills, thus resulting in what is known as Aluminum Fiber Reinforced Concrete (AFRC)^16^. According to Sabapathy et al.^16^, aluminum wire scraps were used as fibers in concrete to evaluate the compressive and tensile strength. A total of three grades of concrete and five different fibre volume fractions were evaluated, and a defined looped shape of aluminum fiber was used assuming that the shape, dimension, and length affect the aluminum performance in concrete. It was concluded that the optimum percentage of the looped aluminum in concrete was the minimum percentage of 0.5% resulting in a compressive strength of 21.8MPa, 33.5MPa, and 44MPa for concrete grades of 20, 30, and 40 respectively. Thus, the realized compressive strength was superior to the original concrete mixes. Alternatively, research was conducted based on the addition of aluminum powder to the concrete mix. Kumar and Ramamurthy^17^ added several grades of aluminum powder in different percentages to mortar mix with a variable water cement ratio of varying. Increasing the dosage of aluminum from 0.25% to 0.50% resulted in a decrease in flexural strength^18^. In masonry blocks requiring low strength concrete, a compressive strength of 17MPa was achieved by the addition of 0.2% aluminum powder and a 0.5 water to cement ratio^19^. One of the major issues observed with the addition of aluminum powder was the chemical expansion of fresh concrete ensuing from the reaction of aluminum with alkaline water forming voids and resulting in poor strength concrete^20^.

Plastic is a material known worldwide, accessible to everyone. It comes in different forms, such as water bottles, boxes, household products, and others as it is used in various fields. The chemical composition of plastics is a long chain of polymers formed from short subunits that are joined together by the polymerization process^21^. This composition affects the characteristics of plastic which include flexibility, durability, and lightweight. This material is cheap and easy to produce, which makes its manufacturing process faster. Due to the high demand for several types of plastics and the single use products produced, quantities produced have increased. This demand lead to an increase in percentages of disposed plastics in landfills. Other than the land pollution caused by plastic wastes when dumped in landfills, plastics produce chemicals during their degradation process that contaminate the soil. To reduce the environmental impact of plastic waste in landfills, the incorporation of plastic to concrete mixture was a suggested solution. Several studies were conducted where plastic was utilized as an additive to concrete or replacement of aggregates. When adding shredded plastic bottles to concrete, it results in what is known fiber concrete. According to Aseel et al.^22^, as the percentages of plastic fibers added to concrete increased, the compressive strength slightly decreased, and the splitting tensile strength increased. These results were confirmed again by Irwan et al.^23^ and specified that the modulus of elasticity decreased as the percentages of plastic added to concrete increased. Foti^24^ has concluded that because of the improved durability of plastic, concrete has increased resistance to the loads resulting in the reduction in cracks.

Glass is a non-crystalline material that is very practical and used worldwide in a versatile manner. In landfills, it constitutes 5.2% of municipal solid wastes (MSW)^25^. Even though a significant percentage of glass containers are being recycled and reused, 7.6 million tons of MSW glass are present in landfills^25^. As a matter of fact, only 34% of waste glass has been recycled^26^; consequently, decreasing the amount of disposed glass waste by using glass waste in concrete can be a good solution. Glass powder (GP) replacement of cement can improve the hardened properties of concrete^26^. Glass is classified as a good additive to concrete without replacing any component of concrete^27^. PG was used as replacement of sand and resulted in a slight increase in the compressive strength^28^. Another study^29^, concluded that a 20% of waste PG replacing sand is an optimum replacement ratio that lead to an improved compressive strength.

The residue left over after the burning of wood and wood products is known as wood ash (WA). About 70% of the wood ash ends up in landfills, where 20% can be used as soil replacement; with a remaining of 10% that is used for various purposes, such as materials for construction, metal recovery, and pollution control. WA has been used as a replacement of several components of concrete. Slump tests performed on fresh concrete mixes with varied percentages of waste WA revealed that mixes containing more wood ash required increased amounts of water to obtain a satisfactory workability^30^. Studies on concrete mixes having substitution of WA for cement concluded that the concrete strength was reduced somewhat as the percentages of WA increased with the optimum percentage to be less than 10%^31^. Several tests were performed on concrete mixes containing WA replacing sand resulted in slump being reduced substantially while at 5% WA as sand substitute lead to a higher compressive strength than the control mix^32^. The same study^32^ indicated that at 5% WA had a lower tensile than control mix concrete while at 20% WA it was greater.

The use of CR recovered from vehicles waste tires in concrete mixes has been explored to elevate the adverse environmental effects caused by concrete on natural resources. Heavy metals and other contaminants in waste tires pose an environmental problem when they are deposited in moist soils in a landfill, causing poisons to leak into groundwater. As a result, structural applications of rubberized concrete have garnered consideration as an efficient means of mitigating environmental concerns. According to Park et al.^33^ the compressive strength declined by 94% as the CR replacement of sand increased. Analogous results were achieved by Wang et al.^34^, they concluded that both the compressive strength of concrete, as well as the flexural strength were reduced as the percentage of crumb rubber increased. Studies by Youssef et al.^35^ obtained that the optimum replacement of sand by CR was 3.5%; and as this value increased up to 9.5% replacement, the compressive strength decreased by about 37%.

CDW dumped in landfill is a permanent and non-biodegradable waste. These wastes can be suitable for a variety of construction jobs, including road construction, curbs, gutters, bridge foundations, shoulders/medians/embankments, and pavement. RCA derived by crushing CDW can replace natural aggregates. According to Novková and Mikulica^36^, RCA derived from CDW can basically replace the natural aggregates of concrete representing around 70% of the total volume of concrete. Thus natural resources are being saved in addition to saving space of landfills and reducing the costs compared with the energy-intensive aggregate exploitations. Other benefits of utilization RCA comprise the decrease of the costs resulted from transferring concrete to the landfill, and moving raw materials to the construction site. All of this in addition to the increase in the life of the landfill resulted from the reduction in the amount of waste dumping. Whereas the useful utilization of RC in concrete is not common, numerous researchers have conducted experimental investigation of concrete mix designs with partial or total replacement of natural aggregates (NA) by RCA. Pavlu et al.^37^ indicated that RCA properties is directly related to the source, quality of the waste material, percentage ratio of components, and the sieve-fraction of aggregates. According to Veriana et al.^38^, the ideal replacement ratio of NA by RCA should range betweem 30% and 50%. Furthermore, Nováková and Mikulica^36^, indicated that a 20% replacement of NA with RCA had no adverse effect on the mechanical and physical properties of concrete and the contrary it yielded an increase of 5.8% in the compressive strength as the result of the existence of residual cement attached to the RCA. According to Tošić et al.^39^, structural concrete incoporating 50% of RCA resulted into an optimum concrete mix based on environmental, economical, and technical aspects. According to Taffese^40^, concrete with 10% RCA had a greater compressive strength compared to NA concrete and same splitting tensile strength with 20% RCA.

## Experimental program

In order to serve the objectives of this research, an experimental program was conducted which included the casting and testing specimens with different variables that were analyzed.

### Concrete mix components

In this study, the ingredients that were utilized in the concrete mixes are:

#### Wood ash (WA)

Wood ash is a residue resulting from the burning of wood and was collected from a local wood bakery. The grain size of wood ash ranges between 0.13 mm to 0.60 mm. The specific gravity of WA was determined to be 1.70.

#### Crumb rubber (CR)

Crumb rubber consists of fine rubber particles ranging in size from 0.075 mm to no more than 4.75 mm. Crumb rubber is generated after shredding the waste tires and removing the steel debris that are found in steel-belted tires.

#### Powdered glass (PG)

Fine crushed glass is a waste glass material obtained from a local waste collector that is crushed in the roller and becomes granulated by sieving. The specific gravity of PG was determined to be 2.45.

#### Plastic bottles (PB)

**Figure 1:**
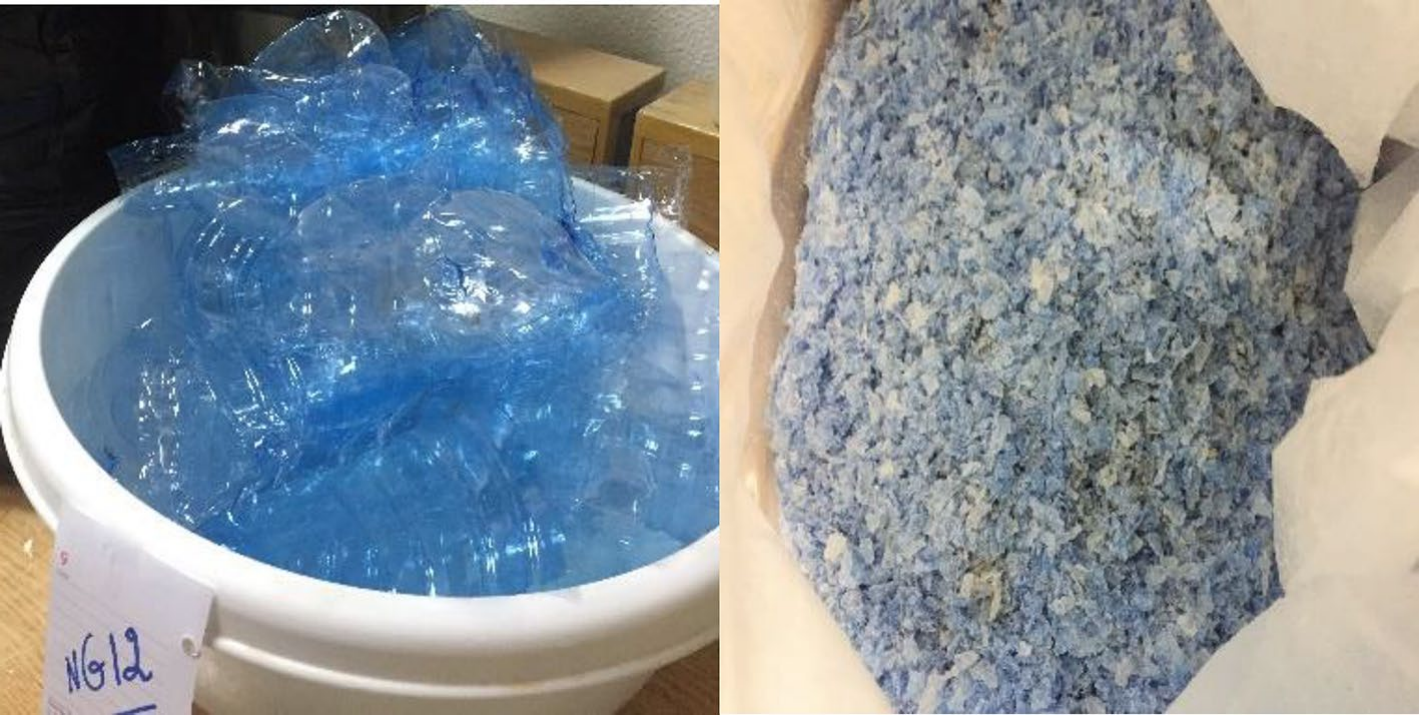
Plastic Bottles Before Shredding and after Shredding.

Plastic bottles (Fig. 1) obtained from a local waste collector were shredded into plastic flakes and were used as an additive to the concrete mix.

#### Aluminum

**Figure 2:**
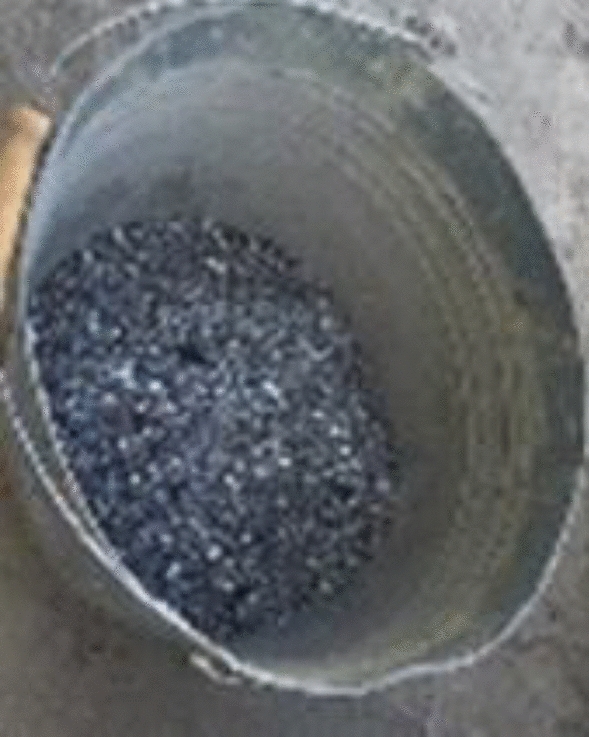
Aluminum Flakes.

Aluminum flakes (Fig. 2) are wastes realized during the drilling of aluminum surfaces during the production of windows and doors.

#### Sand

Natural sand, also known as fine aggregates, is a loose granular substance with grain size of sand ranges between 0.13 mm to 4.75 mm.

#### Medium aggregates (MA)

Medium aggregates any particle greater than 4.75 mm and less than 9.5 mm.

#### Coarse aggregates (CA)

Coarse aggregates, also known as gravel, is any particle equal to 9.5 mm and less than 37.5 mm in diameter.

#### Cement

Ordinary Portland Cement (PAL 42.5) also known as Cement Type I was utilized.

#### Water (W)

Tap water utilized in the mixture.

#### Water reducers

Water-reducing admixtures were incorporated to reduce segregation and improve the flowability of the concrete. In this case, Sika Visco Crete Techno-15 + which is a new generation, powerful, super plasticizing concrete admixture was used.

**Figure 3:**
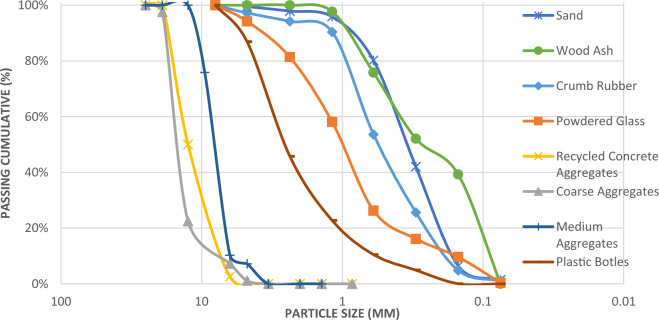
Sieve Analysis Results Concrete Mix Materials.

ASTM C136^41^ was utilized to conduct sieve analysis on the coarse and fine aggregates. The sieve analysis results for the aggregates are displayed in Fig. 3. The particle size distribution of the RCA are similar to that of the CA. The grain size distribution of the aggregates is acceptable within the limits defined by the ASTM C33^42^.

### Concrete mix design

The mix design was performed in a pattern to serve the main scope of this research, which is to establish the most eco-friendly structural concrete mix resulting in an optimum mix incorporating the largest quantity of solid wastes. Five recycled mix (RM) design combinations were examined. Six concrete mixes were tested, one of which was a normal concrete mix that did not contain any waste material, used as a control mix (CM) for comparison and analysis of results. ASTM C192^43^ was utilized in the concrete mixing, casting, curing, and test samples preparation. The first recycled mixture contained all six types of the previously mentioned wastes: powdered glass (PG), crumb rubber (CR), recycled concrete aggregates (RCA), plastic bottles (PB), aluminum flakes (AL), and wood ash (WA). Afterwards, all the remaining tests contained alternating additions of waste to best match the required results. Table 1 summarizes the quantities used in each mix and were utilized to cast 100 × 200 mm cylinders and 150 × 1 50 × 500 mm beams. Tables 1 and 2 summarizes the percentages of recycled materials replacing normal components and the added components.

**Table 1:** Concrete mix composition.

Mix type	Normal concrete components (Kg/m^3^)					Recycled mix components (Kg/m^3^)							Water reducer (Kg/m^3^)	Water/Binder
	Cement	Sand	CA	MA	Water	RCA:CA	CR:Sand	AL	PB	PG:Sand	PG:Cement	WA:Sand		
CM	328	820	508	525	180								1.311	0.55
RM1	311	475	262	525	180	262	16.39	16.39	16.39	163.93	16.39	163.93	1.967	0.55
RM2	313	542	250	542	181	250	13.75	20.83		166.67	16.25	83.33	1.875	0.55
RM3	329	625	250	542	181	250	16.25	4.17	4.17	166.67			1.458	0.55
RM4	328	639	262	525	180	262	16.39		16.39	163.93			0.082	0.55
RM5	348	744	665	445	177	335	12.20		15.24	73.17			0.030	0.51

**Table 2:** Percentages of Concrete Mix Components Replaced and Added.

Mix	% recycled mix components replacing normal components					% added components		
	RCA:CA (%)	CR: Sand (%)	PG: Sand (%)	WA: Cement	WA: Sand	AL	PB	Water reducer (%)
RM1	50	2	20	5%	20%	0.50%	0.50%	0.63
RM2	50	2	20	5%	10%	0.25%	–	0.70
RM3	50	2	20	–	–	0.20%	0.20%	0.52
RM4	50	2	20	–	–	–	0.50%	0.25
RM5	50	2	20	–	–	–	0.50%	0.61

### Concrete mechanical properties experiments

For each mix type, cylindrical specimens 150 mm by 300 mm were cast for each experiment, and the following parameters were obtained according to the applicable ASTM standards:

- Slump test according to ASTM C143^44^.
- Compressive strength at 7, 28, and 90 days according to ASTM C39^45^.
- Splitting tensile strength at 28 days according to ASTM C496^46^.

Based on the best results obtained a full-scale reinforced concrete was cast using the selected recycled mix design.

### Reinforced concrete beams

**Figure 4:**
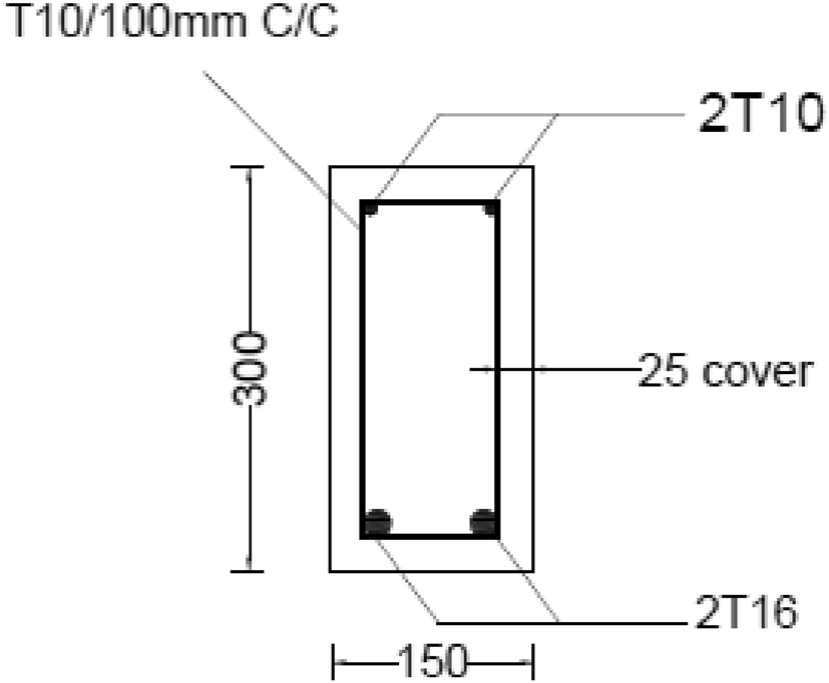
Beam cross section (mm).

**Figure 5:**
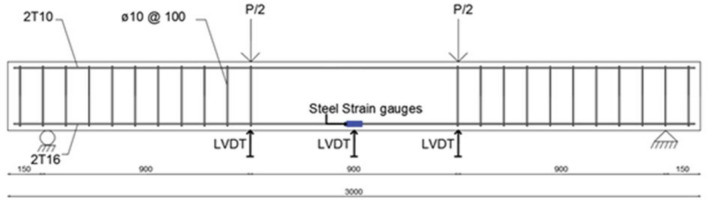
Beam Reinforcement Layout and Test Setup (mm).

The purpose of testing a full-scale beam was to check the performance of the eco-friendly concrete reinforced beam under load and compare it to a regular reinforced concrete beam. The cross section of the beam is shown in Fig. 4 and the reinforcing steel layout and experimental setup are shown in Fig. 5. Testing would be conducted according to ASTM C78^47^.

**Figure 6:**
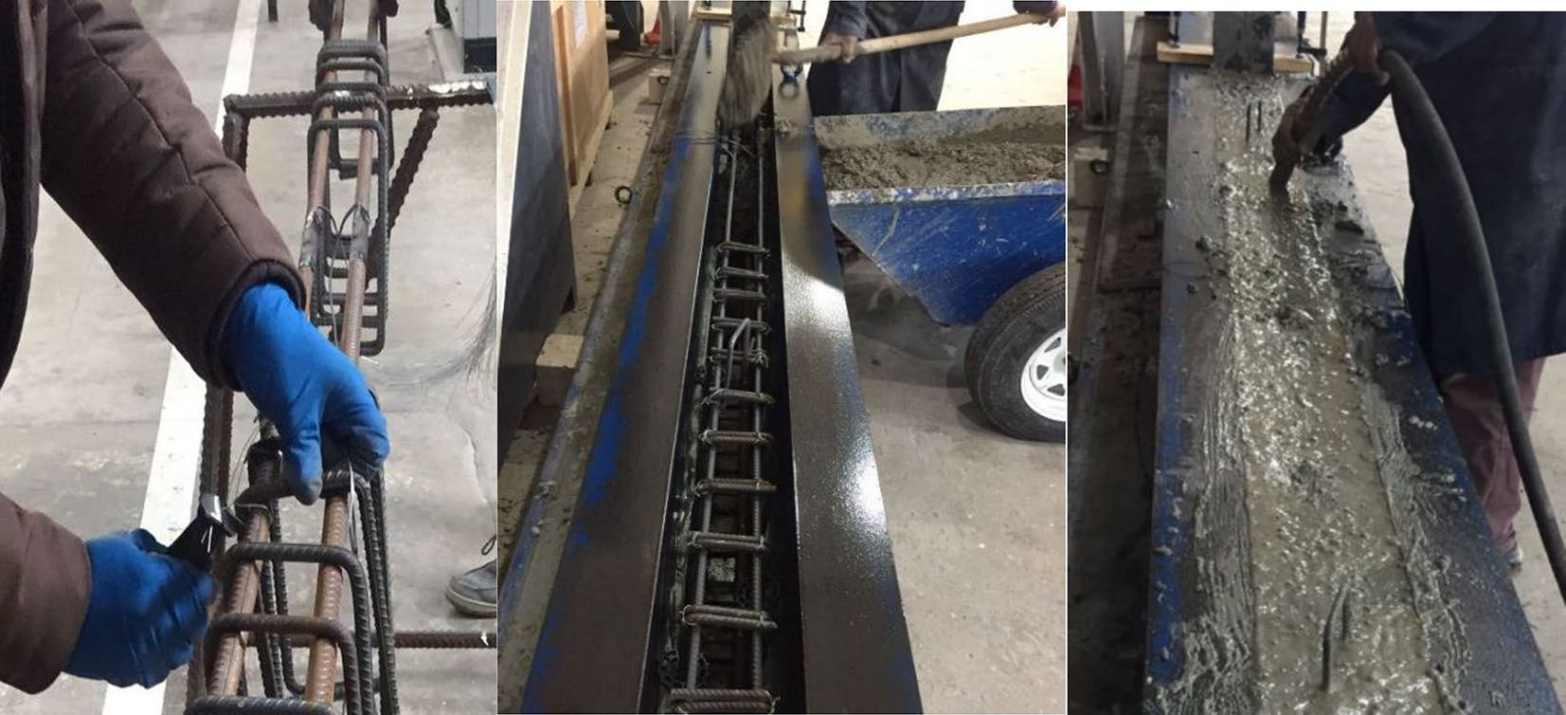
Construction of the Large-Scale Reinforced Beam.

The steps involved in the casting of the beams (Fig. 6) were:

- Strain gauges were placed on the center of each of the two steel bars of T16.
- Stirrups were placed equally on the 4 steel bars used (2T10 top and 2T16 bottom) on the edges leaving the middle third of the beam unreinforced for shear.
- The steel cage was placed in steel mold securing the 25 mm cover.
- Concrete was produced and poured over the setup.
- Mechanical vibrator was used to assure adequate bonding, eliminating any voids, and smoothing the finished surface.
- Concrete was removed at 7 days from the mold and cured for 28 days.

## Results

A total of six concrete mixes were explored to select an optimum eco-friendly concrete mix that is suitable for structural purposes. Each of the recycled mixes had a different percentages of waste components incorporated in the concrete mix. According to ACI 19.2.1.1^48^, concrete mix having wastes products as partial replacement of any component of concrete should have minimum compressive strength of 18 MPa after 28 days of curing.

### Slump results

**Figure 7:**
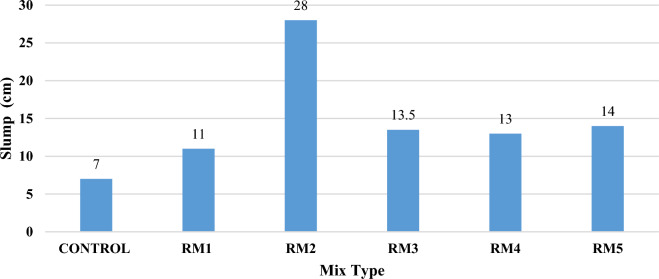
Slump Test.

After each concrete mix, a slump test (Fig. 7) was performed to check the workability and fluidity of concrete. The value of slump test gave a clear view on whether the amount of superplasticizer and water were low, good enough, or in excess. Water reducer amounts were added based on the need of each mix such that all the amounts added were in the specified range per type of water reducer used.

- The CM yielded a slump of 7 cm, the indication of this test shows that the concrete components have good cohesion together. The slump of the control mix was considered as true slump and the value of displacement showed that the concrete mix is of medium workability, between 50 and 100 mm as specified by ASTM C143^44^. This slump showed that the quantity of water reducer added to the mixture was sufficient enough to give good workability of concrete.
- RM1 yielded a slump of 110 mm, it showed good workability of concrete: True–High workability. The water reducer added to water in the presence of all the waste products, displayed good results and good cohesion of fresh concrete.
- RM2 of slump 280 mm, showed over fluidity and workability of concrete. This indicates high water/cement ratio of use of excess water reducer. In this mix, the amount of water was not enough during mixing, so water reducer was added.
- All the remaining slump tests RM3, RM4, and RM5 yielded a slump of 135, 130, and 140 mm respectively. These tests showed true slump with high fluidity, acceptable percentages of water reducer, and good water/binder ratios.

Thus, the slump tests showed that the inclusion of waste products did not affect the workability of concrete; instead, these products blended perfectly with the main concrete components.

### Compressive strength test results

**Figure 8:**
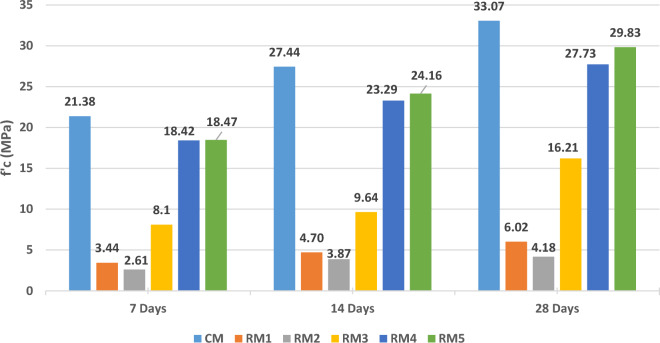
Average Compressive Strength at 7, 14, and 28 days.

The first strength test applied on concrete specimens was the compressive strength. This test gives the results of an essential mechanical property, the maximum compression load that concrete can resist before failing. This test was conducted after 7, 14, and 28 days of concrete curing and results are shown in Fig. 8.

The control specimens and RM1 were cast at the same time; the compressive strength test was performed on this mix first to check its performance. The performance of the mixes was checked, and accordingly other mixes were performed and tested.

In RM1 tests performed very low results of strength were recorded. Even though plastic and aluminum affect negatively the performance of concrete by decreasing its compressive strength, however, and without doubt, the problem was not with this addition; especially that no replacement of main concrete components was done with these wastes.

Based on the results of RM1, to confirm the previous assumption RM2 was performed. After removing plastics completely and reducing the wood ash replacement of sand to 10%, another decrease in the compressive strength was detected. This observation ensured that plastic was not the factor that affected the compressive strength. No conclusion was made at this stage since the results were a bit vague. Not to mention that the slump value of this mix was very high and very workable with no segregation in this mix. The decrease in the compressive strength could be credited to the increase in the amount of water reducer or water/cement ratio.

With both RM1 and RM2 achieved results below structural concrete requirements, RM3 was implemented. In this mix, WA was not used and a total of 4.17 kg/m^3^ of aluminum and the same amount of plastic were added to the mix. By not adding WA, a substantial increase in the compressive strength was observed from average of 3.44 MPa and 2.61 MPa to 8.1 MPa at day 7. This clearly indicated that WA has negatively affected the concrete mix compressive strength. Since its presence in previous mixes was not that effective, it can be concluded that changing the source of wood ash caused a change in its quality, thus affected the concrete mechanical properties.

**Figure 9:**
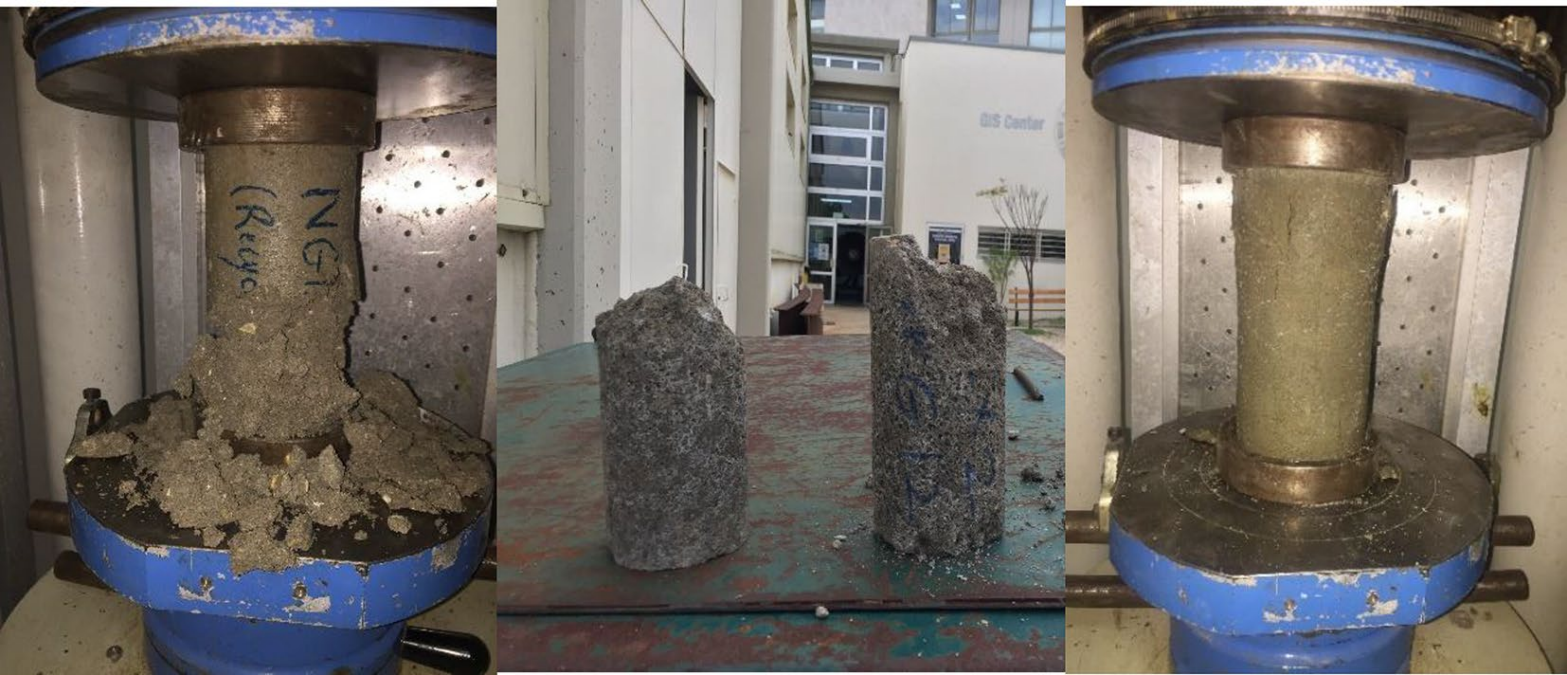
The 7 Day Compressive Strength Test for RM1, RM2, and RM3.

While testing the compressive strength of RM1 and RM2, no cohesion between components of concrete specimens was observed. Instead, when load was applied to the specimens, concrete collapsed into small pieces as shown in Fig. 9 where a different behavior was noted for RM3. Also, while casting RM1, 2, and 3, air bubbles were forming on the surface, without segregation, but volume of concrete increased a bit after pouring it in molds. The increase in volume of concrete can be credited to the chemical reaction of aluminum flakes with alkaline water forming air bubbles. To check for this assumption, concrete composition of RM4 and RM5 did not include any of wood ash or aluminum flakes. No air bubbles and increased volume were detected. Instead, good results with an average 7-day compressive strength above 18 MPa was obtained, where each mix had close results compared to the CM with an average 7-day compressive strength above 21 MPa.

From 7-day results, CM, RM4, and RM5 already achieved the above 18MPa being the minimum 28 days compressive strength at required for structural concrete mix, according to ACI^47^. At 14 days, the compressive strength of RM1 and RM2 were still below the requirements; RM3 displayed a slight increase in compressive strength. However, better promising results were obtained for RM4 and RM5. Figure 8 clearly shows the optimum recycled mix is RM5 displaying the highest compressive strength.

### Splitting tensile strength results

Splitting tensile is a test used on concrete to determine the mechanical behavior of concrete specimen when load is applied vertically along the length of specimen. Due the dismal compressive strength results for RM1 and RM2, this test was conducted at 28 days for CM, RM3, RM4, and RM5.

**Figure 10:**
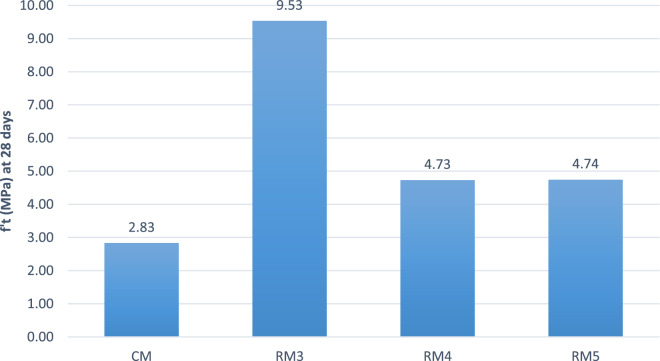
Average Splitting Tensile Strength Results.

The control concrete mix attained an average splitting tensile strength of 2.83 MPa. For RM3, RM4, and RM5, displayed an increased values of tensile strength as shown in Fig. 10. RM3 has displayed the highest average tensile strength of 9.53 MPa. RM4 and RM5 had almost the same average splitting tensile strength. In RM3 mix, both aluminum and PB were added which contributed to this mix yielding the highest splitting tensile result. The addition of only PB to RM4 and RM5 resulted in higher values of tensile strength compared to the control mix, but lower values when compared to RM3. Thus, the tensile strength of recycled concrete mix increased by almost 3.37 times when both aluminum and PB were added to the and increased by 1.67 times when only PB was added.

### Flexure beam results

**Figure 11:**
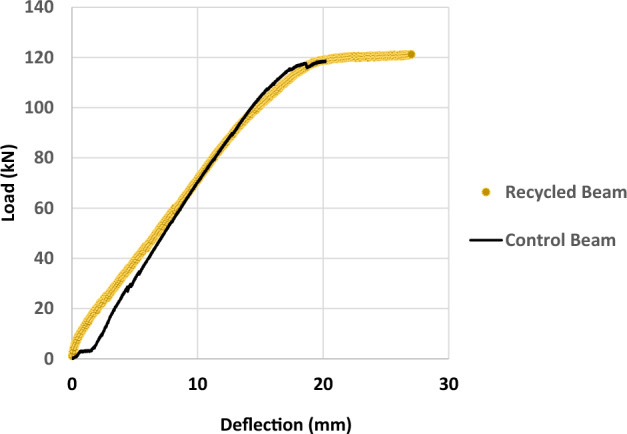
Load Deflection for the Full Scale Beams.

Figure 11 displays the midpoint deflection of the CM and RM5 beams. As shown in Fig. 5, the load was applied over the two points on the beam; as the load increased, the deflection increased gradually with time. Both beams showed linear behavior after the cracking load till reaching the failure load; and the curves had same slope up to 50% of the ultimate load. The deflection at the mid span of the eco-friendly concrete beam displayed a greater flexibility resulting in 27 mm deflection at a maximum applied load of 121.23 kN. For the control beam, the maximum deflection at midspan was 20 mm at a maximum load of 118.72 kN.

**Figure 12:**
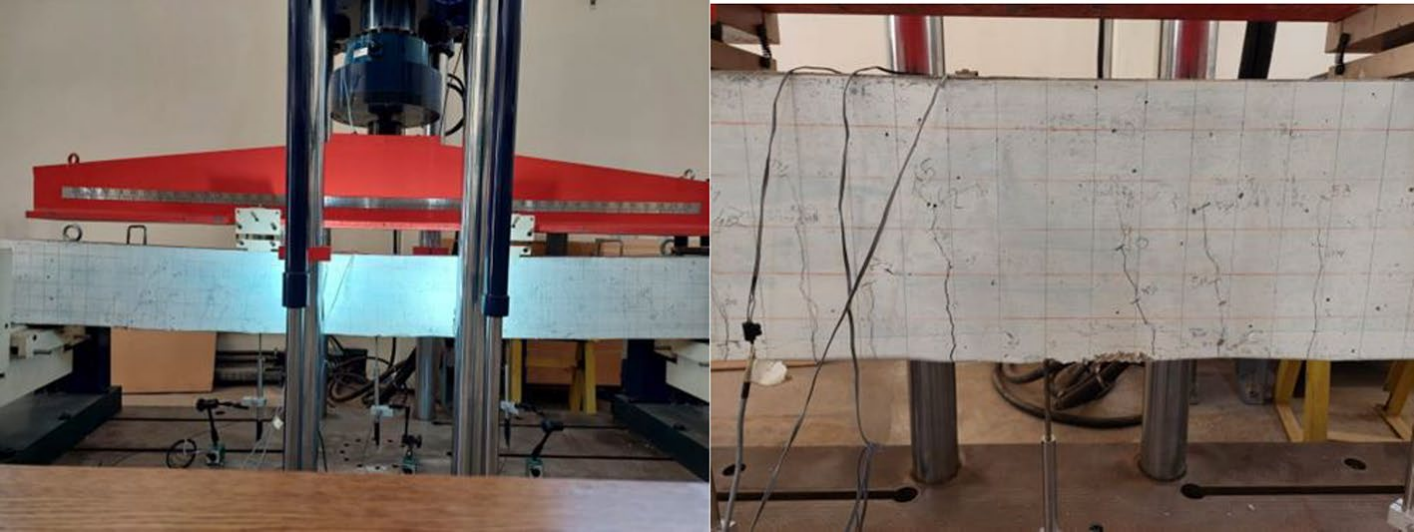
RM5 Beam During Testing and the Development of Crack Patterns.

For both cases, as the load applied increased gradually resulted in serious cracks being detected (Fig. 12). The load kept escalating to reach a time where the load applied was constant. Few fine flexural cracks appeared on the RM5 concrete beam after the load was applied, where the first cracks were recorded at 15 kN. The inclusion of shredded plastic bottles and powder glass in concrete increased increased resistance to the ultimate load before failing.

## Conclusion

The main purpose of this research was to introduce various types of wastes into the concrete mix design leading to the reduction of waste and pollution of the landscape. Some of these wastes were utilized as partial replacement of concrete components where: RCA replaced coarse aggregates, CR and PG replaced sand, and WA replaced sand and cement. Others like PB and aluminum were used as additives to concrete mix.

Based on this study, the followings could be concluded:

- The optimum concrete mix was realized in RM5 which incorporated: 2% CR replacing sand, 20% PG replacing sand, 50% RCA replacing coarse aggregates, and 0.5% PB as additive.
- The results of the slump test showed that none of the recycled mix affected the slump or workability of the concrete mix. Although several previous research showed that WA ash can be incorporated in concrete as 20% replacement for sand and 5% replacement of cement, the use of WA had displayed negative results. Aluminum flakes used in the mixture had a negative effect on concrete. The expansion of concrete volume was detected in mixes containing aluminum, since aluminum chemically reacted with alkaline water producing voids thus causing the expansion of the concrete and introducing voids.
- The RM4 recycled concrete mix containing aluminum, PG, PB, RCA, and CR yielded a maximum compressive strength of 16 MPa at 28 days which indicates that it can be utilized for non-structural applications, since the minimum compressive strength of concrete for structural applications is 18 MPa. On the contrary, the optimum mix (RM5) recorded a compressive strength result of about 30 MPa at 28 days. Even though this strength was less than the CM but a higher tensile strength than that of the CM was obtained. It can also be concluded that the addition plastic bottle fibers and aluminum flakes to concrete increased the tensile strength.
- The RM5 reinforced obviously behaved in a superior fashion when compared to the CM beam. It not only was able to handle a higher load, but it also displayed a superior flexibility capacity in the range of 35% increase in midspan deflection.
- Achieving sustainability was the main objective of this study. A full-scale beam cast from an optimum mix outperformed the control mix beam. Thus, an eco-friendly concrete mix with reduced amounts of natural raw and the incorporation of waste was realized as an initial step towards a sustainable concrete construction cycle.

### Ethics approval

The authors are not misrepresenting research results and are maintaining the integrity of this research and its presentation. This manuscript is only submitted to this journal. The submitted work is original and have not been published elsewhere in any form or language.

## Data Availability

All generated data is presented in the paper.
